# Standardization of T1 measurements with MOLLI in differentiation between health and disease – the ConSept study

**DOI:** 10.1186/1532-429X-15-78

**Published:** 2013-09-11

**Authors:** Toby Rogers, Darius Dabir, Islam Mahmoud, Tobias Voigt, Tobias Schaeffter, Eike Nagel, Valentina O Puntmann

**Affiliations:** 1 Department of Cardiovascular Imaging, King’s College London, London, UK; 2 Clinical Research Europe, Philips Research, London, UK; 3 Department of biophysics and medical engineering, King’s College London, London, UK

**Keywords:** Standardization, T1 mapping, MOLLI

## Abstract

**Background:**

T1 imaging based on pixel-wise quantification of longitudinal relaxation has the potential to differentiate between normal and abnormal myocardium. The accuracy of T1 measurement has not been established nor systematically tested in the presence of health and disease.

**Methods:**

Intra-observer, inter-observer and inter-study reproducibility of T1 imaging was assessed in subjects with left ventricular hypertrophy (LVH, n = 25) or dilated cardiomyopathy (DCM, n = 43). Thirty-eight subjects with low-pretest likelihood of cardiomyopathy served as a control group. T1 values were acquired in a single mid-ventricular short axis slice using modified Look-Locker imaging prior and after the application of gadolinium contrast at 1.5 and 3 T. Analysis was performed with regions of interest (ROI) placed conservatively within the septum or to include the whole short axis (SAX) myocardium.

**Results:**

Intra-observer, inter-observer and inter-study repeated measurements within the septum showed smaller mean differences and narrower 95% confidence intervals than repeated short axis ROI measurements. Native T1 values were higher in septal ROIs compared with SAX values at both field strengths (1.5 T: 976 ± 37 vs. 952 ± 41, p < 0.01; 3 T: 1108 ± 67 vs. 1087 ± 60, p < 0.01). Native T1 values revealed significant mean differences between controls and patients with LVH for both septal (1.5 T: 26 ± 9, p < 0.01; 3 T: 50 ± 13, p < 0.01) and SAX ROIs (1.5 T: 19 ± 11, p < 0.05; 3 T: 47 ± 19, p < 0.05) with greater differences observed at 3 T versus 1.5 T field strength. Native T1 values revealed significant mean differences between controls and patients with DCM for septal ROI (1.5 T: 29 ± 15, p < 0.05; 3 T: 55 ± 16, p < 0.01) at both 1.5 T and 3 T, but only for SAX ROIs at 3 T (49 ± 17, p < 0.01). There were no significant differences in post-contrast T1 values or partition coefficient (λ) between controls and patients.

**Conclusion:**

Conservative septal ROI T1 measurement is a robust technique with excellent intra-observer, inter-observer and inter-study reproducibility for native and post-contrast T1 value and partition coefficient measurements. Moreover, native septal T1 values reveal the greatest difference between normal and abnormal myocardium, which is independent of geometrical alterations of cardiac chamber and wall thickness. We propose the use of native T1 measurements using conservative septal technique as the standardized approach to distinguish health from disease assuming diffuse myocardial involvement.

## Background

Quantification of longitudinal relaxation by T1 mapping provides tissue specific values in line with their composition and magnetic field strength [1]. Interest in T1 mapping has grown exponentially in recent years, with promising yield that it can differentiate between normal and abnormal myocardium in overt cardiac pathologies [2-6]. However, there are several issues pertaining successful translation of this method into clinical practice. Current evidence is derived on the basis of single center studies using a variety of sequences, complex contrast agent injection schemes, pre and post contrast data acquisition algorithms, different doses and types of contrast agent, and non-uniform post-processing approaches [2]. Several studies were based on the classical Look-Locker (or ‘TI scout’) sequence which is widely available due to its accepted application for determination of the null point of the myocardium for LGE imaging [7]. The introduction of modified Look-Locker imaging (MOLLI) offered the advantage of acquiring data at a standstill diastolic point of the heart cycle [8]. Despite ongoing development and novel sequences [9] MOLLI remains the most widely available T1 mapping technique. Whereas the majority of published studies relied on the image acquisition in a single mid-ventricular short axis (mid-SAX) plane, the differences in approaches to post-processing of myocardial T1 are remarkable ranging from segmental approaches using the 16–segment model to the inclusion of the entire myocardium within the mid-SAX plane [3-6,10]. Segmental approaches uncovered significant regional variation of the T1 values with extremes between septal and lateral segments: septal values are higher and show smaller spread of values than lateral [9-11]. Furthermore, lateral segments show greater susceptibility to artifacts. Whether the choice of post-processing matters in discrimination between health and disease given the known regional variation of the T1 values is unknown.

In addition to native and post-contrast myocardial T1 measurements, the calculation of hybrid measures, such as partition coefficient λ have been reported [12,13]. Furthermore, extracellular volume (ECV) can be calculated if the hematocrit is known, using the formula (1-haematocrit)*×λ. Studies varied in their reported T1 values per field strengths, and also in terms of regional variation of extracellular volume fraction [11-13]. Despite some evidence in healthy volunteers and overt cardiac pathologies the robustness of the T1 mapping methodology has not been systematically tested in the presence of health and disease. In particular, whether altered geometric relations of left ventricular (LV) cardiac chamber and wall thickness, commonly encountered in routine clinical patients affects the accuracy and reproducibility of T1 measurements has not been determined.

## Methods

Consecutive subjects referred for clinical Cardiovascular Magnetic Resonance (CMR) were invited to participate in this study. Groups of unrelated subjects were composed to examine the influence of LV chamber size and wall thickness [14]:

1. LV hypertrophy (LVH; n = 25; IVSd ≥ 12 mm, LVPWd ≥ 12 mm, increased indexed LV mass but no increase in LV cavity).

2. Dilated LV cavity (DCM; n = 43; end-diastolic volume index (EDV index) > 100 ml.m^-2^, end-systolic volume index (ESV/index) > 37 ml.m^-2^, reduction in global systolic function (ejection fraction (EF) of < 56%), but no increase in wall thickness).

3. Thirty-eight normotensive subjects with low-pretest likelihood of cardiomyopathy, normal LV volumes, mass, and global systolic function, as well as absence of myocardial Late Gadolinium Enhancement (LGE) and no regular medication served as a control group.

Exclusion criteria for all subjects were the generally accepted contraindications to CMR (implantable devices, cerebral aneurysm clips, cochlear implants, severe claustrophobia) or history of renal disease with a current eGFR < 30 mL/min/1.73 m2. The study protocol was reviewed and approved by institutional ethics committee and written informed consent was obtained from all participants.

### Image acquisition

We integrated native and post-contrast myocardial T1 mapping with MOLLI into our routine imaging protocol for the determination of the underlying etiology of cardiomyopathy as previously described [3]. Studies were performed at either 1.5 or 3 T field strength (Philips Healthcare, Best, The Netherlands) on clinical scanners equipped with advanced cardiac packages and 32-channel coils. After standardized patient specific planning [15], volumetric cavity assessment was obtained by whole-heart coverage of gapless short-axis slices. Thereafter, cine-images of 3 long-axis views (4-chamber, 2-chamber and 3-chamber view) and transverse axial views were acquired. All cine-images were acquired using a balanced steady-state free precession sequence in combination with parallel imaging (SENSitivity Encoding, factor 2) and retrospective gating during a gentle expiratory breath-hold ((TE/TR/flip-angle: 1.7 ms/3.4 ms/60°, spatial resolution 1.8 × 1.8 × 8 mm). LGE imaging was performed in corresponding views in all subjects using a mid-diastolic inversion prepared 2-dimensional gradient echo sequence (TE/TR/flip-angle 2.0 ms/3.4 ms/25°, spatial resolution 1.8 × 2 × 8 mm, with a patient-adapted prepulse delay) 20 minutes after contrast injection (gadobutrol, 0.2 mmol/kg body weight). T1 mapping was performed in a single mid-ventricular short-axis slice prior to administration of 0.2 mmol/kg of gadobutrol and prior to the LGE imaging. Parameters for native and post-contrast MOLLI imaging were identical (FOV 320 × 320; TE/TR/flip-angle: 1.57 ms/3.3 ms/50°, interpolated voxel size 0.9 × 0.9 × 8 mm, phase encoding steps n = 166, HR adapted trigger delay, with 11 (3-3-5) phase sampling arrangements. An adiabatic pre-pulse was used to achieve a complete inversion.

### Image analysis

All routine CMR analysis was performed using commercially available software (ViewForum, Extended Workspace, Philips Healthcare, The Netherlands). Endocardial LV borders were manually traced at end-diastole and end-systole. The papillary muscles were included as part of the LV cavity volume. LV end-diastolic (EDV) and end-systolic (ESV) volumes were determined using Simpson’s rule. Ejection fraction (EF) was computed as EDV-ESV/EDV. All volumetric indices were normalized to body surface area (BSA).

For each subject T1, relaxation values were measured separately by two independent observers. Chosen regions of interest (ROIs) were automatically propagated across all eleven images in the MOLLI sequence with a prior image- co-registration step for motion-correction [3]. Care was particularly taken to avoid ‘contamination’ with signal from the blood pool.

**Figure 1:**
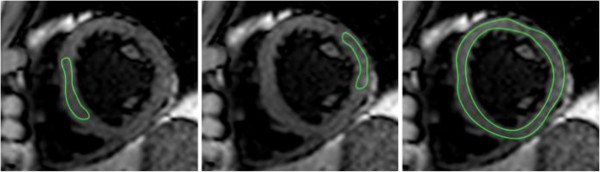
Sample short axis images depicting ROIs places conservatively within the septum, lateral wall or around the whole myocardium.

Two main approaches to place myocardial ROIs within the mid-SAX slice were examined: Septal and SAX myocardial ROI (Figure 1). An additional lateral myocardial ROI was also examined for regional differences in T1 values. In addition to the T1 values of native myocardium and blood pool, we calculated lambda (λ), a marker of interstitial contrast agent accumulation according to the formula λ = [Δ R1myocardium]/[Δ R1bloodpool] pre and post gadolinium contrast where R1 = 1/T1 [11,12].

### Statistical analysis

Statistical analysis was performed using SPSS software (SPSS Inc., Chicago, IL, USA, version 20.0). Departures from normality were detected using Kolmogorov-Smirnov test. Mean differences were examined by one-way and repeated measures ANOVA with Bonferroni post-hoc tests, as appropriate. Comparisons of values between two field strengths, and post-processing approaches were performed using paired and unpaired t-tests and one-way analysis of variance, as appropriate. Agreements between two methods, different observers, and repeated measurements of a single observer were determined by linear regressions, mean differences (bias), 95% confidence interval, and relative differences (mean difference of two techniques/measurements as percentage of their mean value) according to the methods of Bland and Altman. Values are reported as mean±SD and a p-value of less than 0.05 was considered statistically significant.

## Results

Subject characteristics are presented in Table 1. There was a predominance of male subjects in all groups. Patients with DCM were older than normal subjects and patients with LVH. In comparison to controls, patients with LVH had increased LV mass but similar LV volumes and function. Patients with DCM had increased LV volumes with reduced global systolic function.

**Table 1:** Subject characteristics

**Variables**	**Normal**	**LVH**	**DCM**
	**(n = 38)**	**(n = 25)**	**(n = 43)**
Gender (n, % male)	25 (65)	16(67)	29 (67)
Age (years)	49 ± 13	50 ± 13	57 ± 15
Heart rate (bpm)	66 ± 10	65 ± 10	63 ± 9
Body surface area (m^2^)	1.9 ± 0.1	1.9 ± 0.2	1.9 ± 0.2
EDV index (ml.m^-2^)	80 ± 15	72 ± 21	133 ± 37*
ESV index (ml.m^-2^)	32 ± 9	29 ± 12	87 ± 37*
Ejection fraction (%)	61 ± 6	61 ± 12	36 ± 1*3
Mass index (g.m^-2^)	52 ± 15	90 ± 31*	89 ± 29*

*LVH* left ventricular hypertrophy, *DCM* dilated cardiomyopathy, *BSA* body surface area, *EDV* end-diastolic volume, *ESV* end-systolic volume, one way ANOVA, Bonferroni post-hoc tests for the differences from control group, *p < 0.05 is considered significant.

There were significant regional differences between the septal and lateral ROIs for nativeT1, but not for post-contrast values. At both 1.5 T and 3 T field strengths, native septal T1 values were higher than SAX or lateral T1 values (mean of all subjects: T1 values, septal vs. SAX vs. lateral (ms): 1.5 T: 976 ± 37 vs. 952 ± 41 vs. 943 ± 45, p < 0.01; 3 T: 1108 ± 67 vs. 1087 ± 60 vs. 1052 ± 72, p < 0.01). The partition coefficient λ was lower at 1.5 T than 3 T and showed no regional variation at either field strength (mean of all subjects, 1.5 T: septal vs. lateral vs. SAX: 0.42 ± 0.09 vs. 0.43 ± 0.10 and 0.42 ± 0.08; 3 T: 0.47 ± 0.11 vs. 0.46 ± 0.11 and 0.46 ± 0.09). We observed a wide variability of native blood T1 values in all sub-groups with a coefficient of variability (CoV) of 21-28%.

Table 2 lists mean differences (MDs) between septal T1 values and those derived using SAX ROIs or lateral measurements. For both field strengths and all groups, the MDs were greater for lateral measurements than SAX ROIs (p < 0.05 for all). Also, MDs and SDs were greater in both groups of patients than healthy subjects for all measures, and the spread of differences was considerably greater for the DCM group than the LVH group (p < 0.05 for all).

**Table 2:** Mean differences (MD) in T1values between septal and SAX ROIs or lateral measurements (LVH – left ventricular hypertrophy, DCM – dilated cardiomyopathy)

**MOLLI**	**Native T1 values (MD ± SD)**		**Post-contrast T1 values (MD ± SD)**		**Lambda (MD ± SD)**	
**1.5 T**	Septal vs. SAX	Septal vs. Lateral	Septal vs. SAX	Septal vs. Lateral	Septal vs. SAX	Septal vs. Lateral
Normal	16 ± 11	21 ± 43	6 ± 14	11 ± 28	0.002 ± 0.03	0.003 ± 0.06
LVH	20 ± 21	29 ± 49	11 ± 19	13 ± 39	0.01 ± 0.04	0.02 ± 0.09
DCM	28 ± 31	50 ± 64	19 ± 22	25 ± 55	0.02 ± 0.04	0.02 ± 0.11
**3 T**						
Normal	21 ± 22	46 ± 46	10 ± 18	13 ± 20	0.002 ± 0.08	0.02 ± 0.09
LVH	16 ± 41	40 ± 37	17 ± 26	16 ± 37	0.01 ± 0.06	0.01 ± 0.10
DCM	26 ± 49	55 ± 92	24 ± 38	27 ± 43	0.02 ± 0.10	0.02 ± 0.12

For both field strengths and all groups, the MDs were greater for lateral measurements than SAX ROIs (p < 0.05 for all).

**Figure 2:**
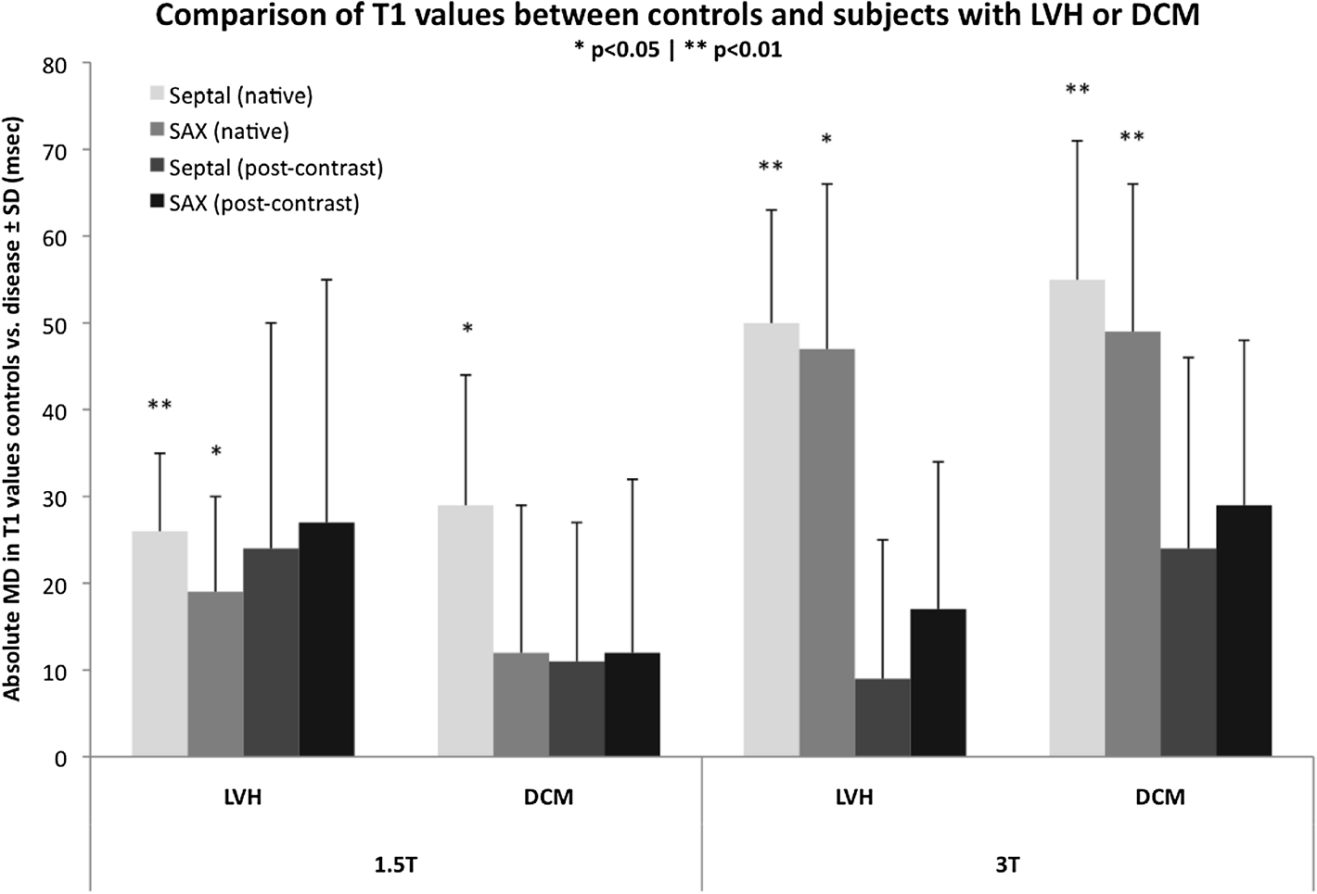
**Comparison of T1 values between controls and subjects with LVH or DCM at 1.5 T and 3 T field strengths.** Results are expressed as absolute mean difference (MD) ± SD (ms). Differences with statistical significance are identified by * p < 0.05 and ** p < 0.01.

Table 3 lists the effective differences between controls and patient groups. Native T1 myocardium revealed significant difference between controls and patients (p < 0.05) for septal and SAX approach, with greater differences observed at 3 T compared with 1.5 T field strength. Post-contrast measurements at either field strength showed no significant difference between controls and patient groups within the current cohort size. The partition coefficient λ showed no significant difference between the groups for either field strength. Figure 2 summarizes the mean differences for both 1.5 T and 3 T field strength.

**Table 3:** Detecting the difference between controls and patients using septal and SAX myocardial ROI, sequence, and field strengths

	**Native T1 values**		**Post-contrast T1 values**		**Lambda**	
	**(MD ± SD)**		**(MD ± SD)**		**(MD ± SD)**	
**LVH**						
	Septal	SAX	Septal	SAX	Septal	SAX
1.5 T	26 ± 9**	19 ± 11*	24 ± 26	27 ± 28	0.02 ± 0.04	0.03 ± 0.03
3 T	50 ± 13**	47 ± 19*	9 ± 16	7 ± 17	0.07 ± 05	0.04 ± 0.04
**DCM**						
	Septal	SAX	Septal	SAX	Septal	SAX
1.5 T	29 ± 15*	12 ± 17	11 ± 16	12 ± 20	0.01 ± 0.03	0.01 ± 0.03
3 T	55 ± 16**	49 ± 17**	24 ± 22	29 ± 19	0.02 ± 0.03	0.01 ± 0.04

Absolute mean difference (MD) and standard errors (SE) between controls and separate patient groups are derived with Student *t*-test, p-value < 0.05*, < 0.01**.

**Figure 3:**
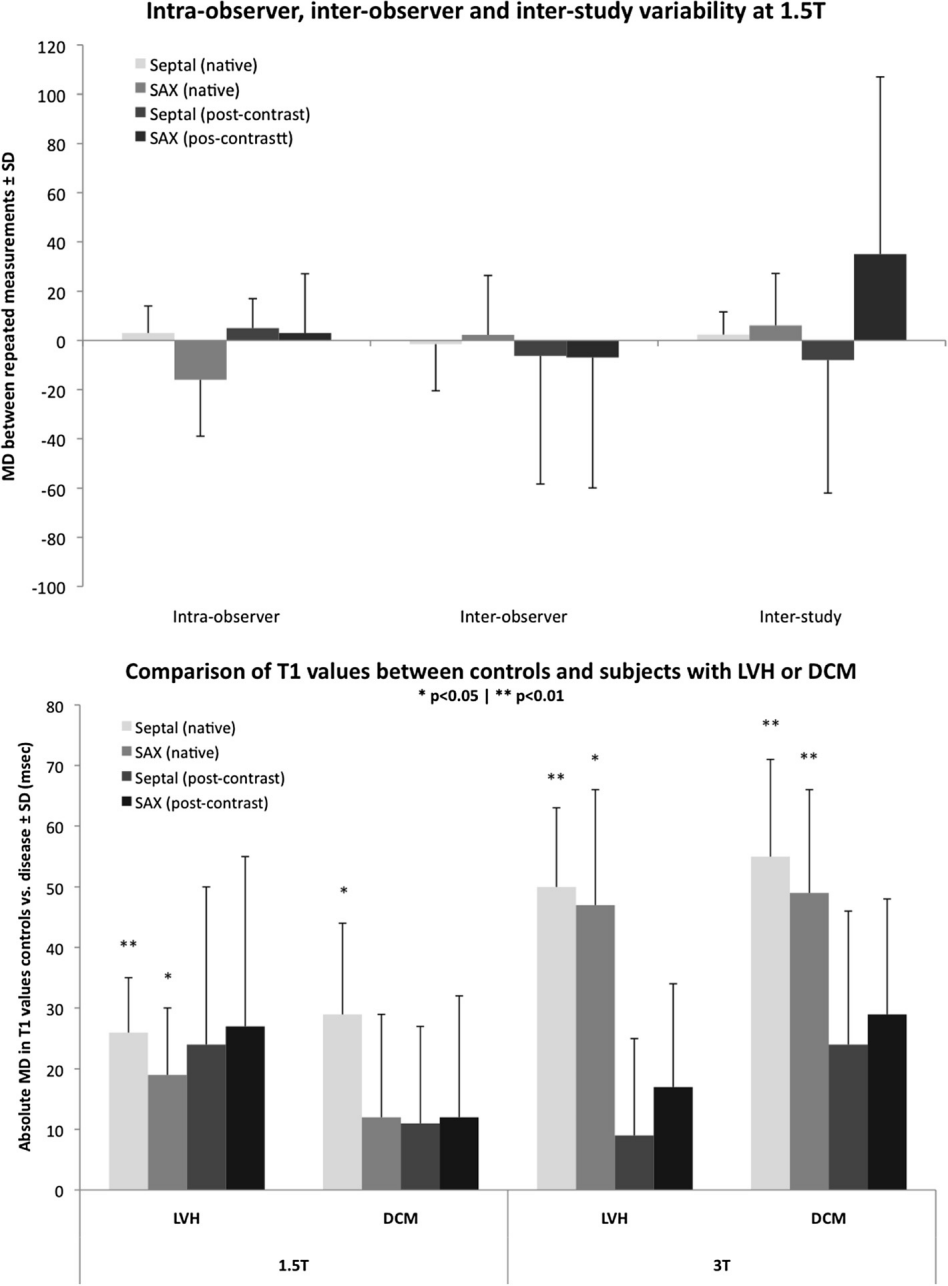
**Intra-observer, inter-observer and inter-study variability at 1.5 T and 3 T field strength.** Results are expressed as mean difference (MD) between repeated measurements ± SD (ms).

Table 4 lists results of intra- and inter-observer reproducibility and inter-study reproducibility of measurements. Intra-observer reproducibility for both field strengths septal measurements showed smaller MDs and narrower 95% confidence intervals. Compared to post-contrast values and partition coefficient, native T1 values measured in the septum showed superior intra-observer reproducibility at both field strengths. Figure 3 summarizes mean differences and 95% confidence intervals for all three reproducibility tests for septal vs. SAX native and post-contrast T1. For both field strengths septal measurements show smaller MDs and narrower 95% confidence intervals and higher reproducibility compared with the SAX approach.

**Table 4:** Intra-observer, inter-observer and inter-study reproducibility of measurements for all subjects

**Intraobserver**	**1.5 T**		**3 T**	
	**Septal**	**SAX**	**Septal**	**SAX**
**Native T1**				
MD ± SD (ms)	3 ± 11	-16 ± 23	3 ± 13	-6 ± 16
CoV (%)	1.1	2.3	1.2	1.5
Agreement (r)	0.84**	0.72**	0.87**	0.74**
**Post-contrast T1**				
MD ± SD (ms)	5 ± 12	3 ± 24	-6 ± 15	-3 ± 13
CoV (%)	3.1	6.6	3.2	3.2
Agreement (r)	0.78**	0.53*	0.79**	0.62**
**Lambda**				
MD ± SD	0.014 ± 0.009	-0.017 ± 0.013	0.017 ± 0.002	0.019 ± 0.019
CoV (%)	4.1	5.2	5.1	5.9
Agreement (r)	0.81**	0.69**	0.78**	0.61**
**Interobserver**	**1.5 T**		**3 T**	
	**Septal**	**SAX**	**Septal**	**SAX**
**Native T1**				
MD ± SD (ms)	-1.5 ± 19	2.3 ± 24	0.3 ± 15	-2.9 ± 49
CoV (%)	4.3	6.9	1.4	2.7
Agreement (r)	0.89**	0.73**	0.93**	0.75**
**Post-contrast T1**				
MD ± SD (ms)	-6.3 ± 52	-6.9 ± 53	6.2 ± 71	-8.4 ± 79
CoV (%)	4.3	7.1	2.8	3.3
Agreement (r)	0.79**	0.69**	0.81**	0.54**
**Lambda**				
MD ± SD	0.013 ± 0.019	-0.017 ± 0.002	0.019 ± 0.008	0.011 ± 0.003
CoV (%)	7.2	7.9	9.1	11.7
Agreement (r)	0.71**	0.59**	0.68**	0.53**
**Interstudy**	**1.5 T**		**3 T**	
	**Septal**	**SAX**	**Septal**	**SAX**
**Native T1**				
MD ± SD (msec)	2.4 ± 9.2	6.1 ± 21	-1.5 ± 12	4.9 ± 18
CoV (%)	1.2	4.5	3.6	8.4
Agreement (r)	0.92**	0.78**	0.95**	0.86**
**Post-contrast T1**				
MD ± SD (ms)	-8 ± 54	35 ± 72	19 ± 65	31 ± 62
CoV (%)	9.0	12	12	15
Agreement (r)	0.62**	0.45*	0.55**	0.41**
**Lambda**				
MD ± SD	0.017 ± 0.021	0.021 ± 0.029	0.016 ± 0.018	0.019 ± 0.035
CoV (%)	4.2	6.1	3.5	7.8
Agreement (r)	0.79**	0.69**	0.82**	0.73**

Data is expressed as mean ± SD, *CoV* coefficient of variability, r = Pearson correlation coefficient for agreement between two measurements, p-value < 0.05*, < 0.01** for significant association.

Inter-study reproducibility was performed in subgroups of normal subjects (n = 12), patients with dilated cavity (n = 6) and subjects with increased LV wall thickness (n = 5) who underwent a second CMR study within up to 1-month interval with no interim change in clinical status or medication. Septal and SAX approaches showed excellent reproducibility and agreements in both field strengths whereas post-contrast T1 measurements showed high variability irrespective of sequence or field strength. Reproducibility of partition coefficient λ was inferior to that observed with native T1 measurements.

## Discussion

Results of our study reveal that the choice of septal vs. SAX approach matters with respect to reproducibility of measurements and detection of effective difference between health and disease assuming diffuse myocardial involvement. We demonstrate that using a conservative septal approach is the more reproducible within and between observers compared with evaluation of the complete SAX slice. Our results also reveal that using septal approach is also most robust for measurements in repeated studies within the same subject. We further show that septal T1 measurements in native myocardium reveal the greatest difference between health and disease, irrespective of the field strength or geometrical changes due to cardiac pathology. Results of our study provide a proof of concept that T1 measurement derived conservatively within the septal myocardium should be adopted as the standardized approach in measurement of T1 values when assuming diffuse involvement. Using this approach, we further propose the native T1 as the best discriminatory measure between health and disease.

Several studies report on excellent reproducibility of T1 measurements prior to or after administration of the contrast agent [8,9,12,13]. These studies however employed a variety of sequences and sampling approaches and predominantly focused on a healthy population. A single previous study examined the reproducibility in health and disease [16], based on calculation of lambda and ECV. Our study concords with many of the reported findings including higher values found in patients and in terms of variability of measures. Our study expands on these previous findings, by including the native T1 values, and a head-to head comparison with the postcontrast T1 and lambda values, performed at both field strengths. Furthermore, we specifically placed our focus on the accuracy of post-processing in relation to altered chamber dimensions and wall thickness [14], as well as in light of known regional variations of T1 values [8,9]. We show that native T1 measurements using septal or SAX approach are robust with an overall good intra-observer and inter-observer reproducibility and irrespective of the field strength. We further report that using conservative septal approach native T1 values outperform the postcontrast and hybrid measures in reproducibility and effect size. A few considerations may explain this observation. First, we reproduce previously reported regional variations with a signal gradient in T1 values between the septal and the lateral wall, irrespective of the field strength [9,13]. The SAX approach is a combination of all T1 values observed within the whole SAX slice and as such also includes the lateral T1 values, which show a significantly higher variability than the septum. The observed regional differences unlikely represent a true difference in tissue composition (i.e. a true tissue dependent difference in longitudinal relaxation), in accord with several previous studies demonstrating homogeneity of diffuse myocardial fibrosis in subjects with DCM [17,18]. Instead, the observed differences are likely related to a number of confounding factors, including magnetic susceptibility artifacts, measurement errors and issues pertaining receiver coil sensitivity in SENSE imaging [13,19]. The sampling within the lateral wall suffers with high likelihood from inclusion of voxels outside of the true myocardium or at the very least, the partial volumes of straddling voxels on the myocardium-blood or myocardium-lung interfaces resulting in a mixture of T1 values. Additionally, the signal gradient between septal and lateral myocardium due to greater distance from the receiver coil elements for the latter has been long recognized. The difficulty in drawing ROIs within the lateral wall renders the SAX approach less robust and the benefit of its inclusion into the T1 sampling questionable.

We also show that whereas post-contrast measurements are relatively observer-independent, these measurements are less reliable when cross-correlated between different studies within the same subject. These findings concord with the previous postulations indicating the relatively dynamic post-contrast evolution of T1 myocardial values and an ~150-200 ms change within the usual time-frame between 10–25 minutes when most of contrast-enhanced studies are performed [20]. Typical timing of our post-contrast T1 acquisition coincided with the LGE studies, therefore, we observed that the discrepancy in the post-contrast T1 values is inherently more common in those cases where a shorter second study was focused on the acquisition of T1 mapping dataset. The partition coefficient λ, which also accounts for the differences between native and post-contrast acquisitions, is comparably more robust than simple post-contrast T1 measurements for the inter-study comparisons. Our study showed no significant regional differences in λ, however it revealed a considerable difference in values between 1.5 T and 3 T, as previously also shown by Sharma et al. [21]. A more heterogeneous representation of subjects in the current study and inclusion of more pathological cases in the 3 T cohort may also account for this observation.

The ultimate goal of T1 imaging is a robust clinical application for differentiation between normal and abnormal myocardium [3-5,22-25]. Contrasting the previous studies, we examined the value of T1 measures with respect to commonly occurring patterns of cardiac pathology presenting in a real clinical practice [26]. Within subject comparison of sampling approaches and the use of post-contrast and hybrid measures reveals that conservative septal T1 measurements provide the greatest measurable difference between health and disease when assuming diffuse involvement. Detection of tissue differences is most effective using a native T1 measurement with a standardized approach of a septal approach, which is independent of changes of left ventricular thickness due to myocardial disease process, such as hypertrophy or cavity dilatation. In the current study particular attention was placed on the motion correction as well as the placement of the ROIs conservatively within the myocardium to avoid contamination with the blood pool. Whereas increased wall thickness clearly simplifies ROI placement within the myocardium, the likelihood of measurement of error in the thinned dilated myocardium is considerably higher and further compromised by difficulty in breath-holding resulting in respiratory motion. We also show that T1 mapping using MOLLI sequence is realistic and easily incorporated into routine clinical CMR examination protocols.

## Limitations

Several limitations pertain this study. The small sample size may limit the generalization of present findings as well as assessment of multifactorial influences on T1 measurements. For routine clinical application of T1 mapping, studies with larger subject samples are required to determine normal standard ranges for T1 values. Clinical outcome data must then be assembled to confirm clinical relevance of these measurements. T1 sampling in the septum of a single short axis slice is based on assumption that it is representative of a diffuse myocardial process. Future studies using whole-heart acquisitions with lower susceptibility to regional variations in T1 values are needed to determine and understand the relevant regional variation. Inferior inter-study reproducibility of the partition coefficient λ may be explained by changes in hematocrit between scans. We did not collect blood samples for determination of hematocrit from these healthy volunteers. As this study was based on sampling from clinical environment, individuals have not undergone parallel assessments on both field strengths, thus, our study lacks the comparative data between the two field strengths. Finally, T1 values obtained by our group in control and patient subgroups are relatively lower than those reported by other groups [6,13], indicating the need for cross-center and cross-vendor comparison of sequences.

## Conclusions

Conservative septal ROI T1 measurement is a robust technique with excellent intra-observer, inter-observer and inter-study reproducibility for native and post-contrast T1 value and partition coefficient measurements. Moreover, native septal T1 values reveal the greatest difference between normal and abnormal myocardium, which is independent of geometrical alterations of cardiac chamber and wall thickness. We propose the use of native T1 measurements using conservative septal technique as the standardized approach to distinguish health from disease assuming diffuse myocardial involvement. The superior performance is afforded by the ability to eliminate measurement errors, sampling of voxels straddling the myocardial-blood pool interface, and reduced signal in the lateral wall.

## Competing interests

The authors declare that they have no competing interests.

## Authors’ contributions

TR, EN, VP – study design, TR, DD, IM, VP – data acquisition, analysis, manuscript preparation. TV, TS, EN, VP – sequence validation. All authors read and approved the final draft of this manuscript.
